# Silencing an insulin-induced lncRNA, LncASIR, impairs the transcriptional response to insulin signalling in adipocytes

**DOI:** 10.1038/s41598-019-42162-5

**Published:** 2019-04-04

**Authors:** Ufuk Degirmenci, Jia Li, Yen Ching Lim, Diana Teh Chee Siang, Shibo Lin, Hui Liang, Lei Sun

**Affiliations:** 1 Cardiovascular and Metabolic Disorders Program, Duke-NUS Graduate Medical School, 8 College Road, Singapore, 169857 Singapore; 2 Institute of Molecular and Cell Biology, Agency for Science, Technology and Research, 61 Biopolis Drive, Proteos, Singapore, 138673 Singapore; 3 Department of Biological Sciences, National University of Singapore, Singapore, 117558 Singapore; 4 Department of General Surgery, the First Affiliated Hospital of Nanjing Medical University, Nanjing, 210029 China

## Abstract

Long noncoding RNA(lncRNA)s are new regulators governing the metabolism in adipose tissue. In this study, we aimed to understand how lncRNAs respond to insulin signalling and explore whether lncRNAs have a functional role in insulin signalling pathway. We treated primary adipocyte cultures with insulin and collected RNA for RNA-sequencing to profile the non-coding transcriptome changes, through which we identified a top Adipose Specific Insulin Responsive LncRNA (LncASIR). To determine its biological function, we knocked down LncASIR using dcas9-KRAB, followed by RNA-seq to examine the effect on insulin-induced gene expression program. We identified a set of lncRNAs regulated by insulin signalling pathway. LncASIR is transcribed from a super enhancer region and responds robustly to insulin treatment. Silencing LncASIR resulted in an impaired global insulin-responsive gene program. LncASIR is a novel and integral component in the insulin signalling pathway in adipocytes.

## Introduction

Insulin is an essential hormone in maintaining glucose homeostasis. While most mammalian cells express insulin receptors, essential targets for insulin are metabolic organs such as muscle, liver and adipose^1^. Insulin binds to insulin receptor to trigger insulin signalling cascade that leads to glucose uptake and gene program changes for altered lipid and carbohydrate metabolism. Downstream of insulin signalling pathway includes immediate and long-term responses^2^. Insulin signalling leads to autophosphorylation of IRS1 that activates PI3K-Akt pathway by recruiting and activating PI3K to its substrate PIP2. PIP2 is converted to PIP3 to recruit and activates PDK-1 which in return phosphorylates Akt. Phosphorylated Akt mediates most of the downstream effects upon acute insulin stimulation. In a prolonged insulin exposure, transcription of Fabp4, Glut4, Srebp1c and other lipogenic genes are increased to enhance energy storage^3,4^. However, prolonged hyperinsulinemia can lead to insulin resistance^5^.

Response to insulin is often impaired in adipose tissue during obesity and Type 2 Diabetes(T2D)^6^. Adipocytes are overloaded with lipids during obesity and develop enlarged cells, referred to as hypertrophy^7^. The adipose tissue also develops chronic inflammation due to macrophage infiltration^8^. Immune response triggers enhanced phosphorylation of Serine/Threonine residues in insulin receptor substrate 1 (IRS-1) by JNK^9^ instead of the insulin receptor tyrosine kinase that phosphorylates IRS-1^10^. Serine/Threonine phosphorylation of IRS-1 decreases PI3K activity and render adipocytes insulin resistant^11–13^. A detailed understanding of insulin signalling pathway is a prerequisite for developing new therapeutic strategies for obesity-related type 2 diabetes. However, there is a limited number of studies on the role of long noncoding RNAs (lncRNAs) in insulin signalling pathway.

LncRNAs are novel regulators of gene expression^14,15^. LncRNAs often fine-tune the expression of mRNAs so that cells can respond to environment robustly^16–18^. The expression of lncRNAs, in comparison with protein-coding mRNAs, is more tissue and developmental stage specific^19–21^. LncRNAs can be divided into multiple categories based on nearby coding gene such as intergenic, intragenic, antisense, bidirectional lncRNAs or based on genomic loci they are transcribed from: promoter-related, enhancer RNA, super enhancer lncRNA, intergenic and ultra-conserved lncRNA^22–24^. lncRNAs’ localization in the cell can provide clues on their functions^25^. LncRNAs located in cytosol could affect mRNA stability and translational efficiency^14^ by acting as sponges for miRNAs^26^ or direct pairing with mRNA to modulate their stability or translational efficiency^27^; lncRNAs located in the nucleus often affect chromosomal structure/conformation to regulate gene transcription^28–30^.

The number of identified LncRNAs is snowballing, yet our knowledge of lncRNAs’ functions is still very limited^14^. In adipose tissue, studies from others and our group have revealed the functions of a few lncRNAs^31–39^. Blnc1 drives thermogenesis by binding EBF2 to form a feed-forward loop on brown and beige adipogenesis^32–34^, LncBATE1 interacts with hnRNPU to regulate brown adipogenesis^35^. LncBATE10 decoys Celf1 from PGC1alpha mRNA to enhance Pgc1a expression to promote brown/beige fat programme^36^. Lnc-leptin, an adipocyte-enriched enhancer lncRNA, controls the expression of leptin protein in adipocyte through stabilizing enhancer-promoter looping^37^. During adipocyte differentiation, ADINR transcriptionally activates C/EBPalpha^38^. LncRNA Gm15290 sponges miR-27b to promote lipid accumulation in adipocytes by enhancing PPARγ mRNA stability^39^. Even so, the function of lncRNAs in insulin signalling pathway remains largely unexplored.

Several lncRNAs have been reported to regulate insulin signalling pathway in other cell types. The lncRNA, CRNDE, is downregulated upon insulin treatment in cancer cells^40^. LncSHGL is identified in the liver as a negative regulator for gluconeogenesis and lipogenesis^41^. MEG3 promotes insulin resistance in the liver by increasing FoxO1 expression^42^. Two β−islet enriched lncRNAs, βlinc2 and βlinc3, increased β-cell apoptosis without affecting insulin secretion^43^. In this study, we investigated the lncRNAs that regulate insulin response in adipocytes. Using RNA-sequencing, our study revealed 343 lncRNAs responsive to insulin treatment and identified a top responding lncRNA, Adipose Specific Insulin Responsive lncRNA(lncASIR), which was required for a full insulin-induced gene program in adipocytes.

## Results

### Insulin treatment in adipocytes activates metabolic program

**Figure 1:**
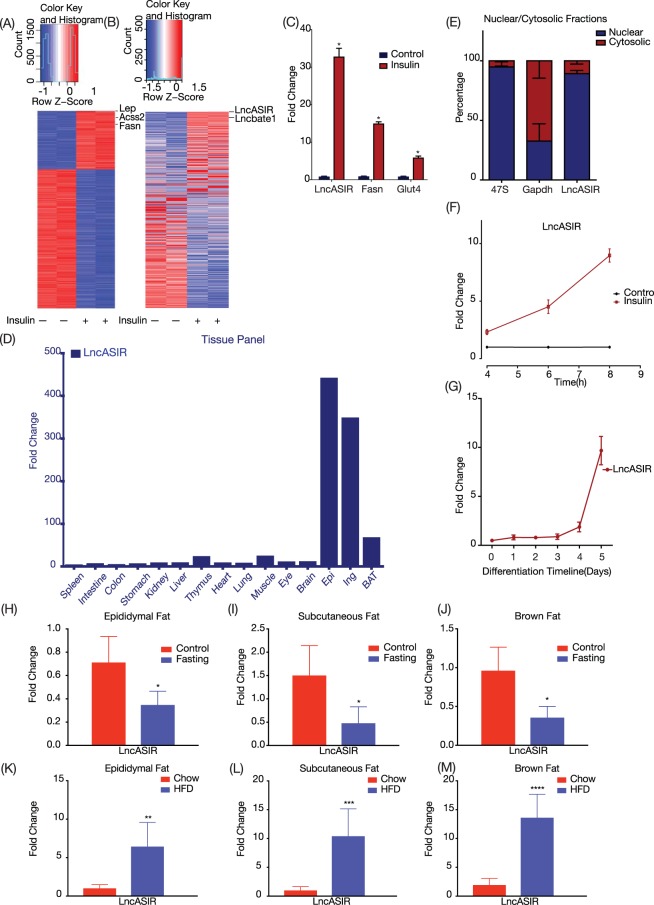
Identification and characterization of lncASIR (**A**). Heatmap of RNA-sequencing from differentially expressed mRNAs in insulin treated primary mature adipocytes, Lep, Acss2 and Fasn marked for reference. (**B**) Heatmap of RNA-sequencing for differentially expressed lncRNAs from same dataset as 1A. LncASIR and lncbate1 is labelled on the side of heatmap for reference. (**C**) Validation of RNA-seq with Quantitative RT-PCR from insulin treated adipocytes for LncASIR, Fasn and Glut4. (**D**) Quantitative RT-PCR from tissue panel for LncASIR expression, RPL23 used as to calculate ΔΔCT. (**E**) Cellular fractionation from adipose tissue for localization of LncASIR (n = 3). 47S and Gapdh used as nuclear and cytosolic control respectively. (**F**) Expression of LncASIR during insulin treatment time course in mature adipocytes (n = 6). (**G**) Expression of LncASIR during adipocyte differentiation time course by Quantitative RT-PCR (n = 4). (**H**–**J**) Expression of LncASIR by Quantitative RT-PCR in 8 weeks old mice fat pads upon overnight fasting (n = 5). (**K**–**M**) LncASIR expression in high fat diet mice. Mice were fed HFD starting from 3 weeks old for 3 months (n > 6). Unpaired t-test was used to calculate p-value. *P < 0.05. Error bars are SEM. Graphs have been drawn using Prism 8.

Preadipocytes were harvested from the stromal vascular fraction of inguinal white fat and cultured to confluence, followed by differentiation for 6 days. Mature adipocytes were fasted for 6 hours with FBS free media and treated with insulin for 8 hours. RNA was collected for sequencing, which generated 58–79 million reads per sample (Figure S1A). As expected, transcriptome analysis showed that several well known insulin-responsive markers such as Leptin(Lep), Acyl-CoA Synthetase Short Chain Family Member 2(Acss2), Fatty Acid Synthase (Fasn) were indeed up-regulated by insulin (Fig. 1A) and pathway analysis demonstrated enrichment for metabolic pathways such as carbon metabolism, biosynthesis of amino acids, steroid biosynthesis and glycolysis/gluconeogenesis.

### RNA-seq data revealed a set of insulin-responding lncRNAs in adipocytes, including LncASIR

The transcriptome analysis revealed a list of lncRNAs that were regulated by insulin signalling. Top lncRNA was referred as Adipose Specific Insulin Responsive lncRNA (lnc-ASIR), which is the most regulated lncRNA (Fig. 1B). It is noteworthy that insulin regulates the expression of LncASIR more dramatically than its well established downstream effectors such as Glut4 and Fasn (Fig. 1C). We examined the expression of LncASIR across different tissues and found a clear enrichment in adipose tissues, particularly in the White Adipose Tissue(WAT)s (Fig. 1D). To determine its cellular localization, we isolated the cytosolic and nuclear fractionation from primary adipocyte cultures for real-time PCR analysis. Pre-47S and GAPDH used as nuclear and cytosolic controls, respectively. The majority (~80%) of LncASIR was located in the nuclear fraction (Fig. 1E). When lncASIR was overexpressed, there was no difference in insulin downstream gene expression (Supplementary 4).

We conducted real-time PCR to confirm its response to insulin and found LncASIR’s expression is increased up to 10 fold compared with control cells without insulin treatment (Fig. 1F). Additionally, LncASIR increases drastically during adipocyte differentiation (Fig. 1G).

Since insulin is a master regulatory hormone responsive to different energy status, we tested whether LncASIR is regulated at energy deficit and surplus conditions *in vivo*. LncASIR decreased by 50% after overnight fasting in iWAT, eWAT and Brown Adipose Tissue(BAT) (Fig. 1H–J). On the other hand, LncASIR showed more than 10-fold increase at high fat diet condition (Fig. 1K–M). High fat diet (HFD) mice were fed with rodent 60%kcal fat, 20%kcal protein and 20%kcal carbohydrate food starting from 3 weeks old for 3 months and mice were sacrificed together with chow diet controls. Because insulin level is reduced during fasting but increased at HFD condition, these *in vivo* data are consistent with the cell culture data (Fig. 1C) and demonstrate that LncASIR is a *bona fide* player downstream the insulin pathway.

### LncASIR is an enhancer lncRNA

**Figure 2:**
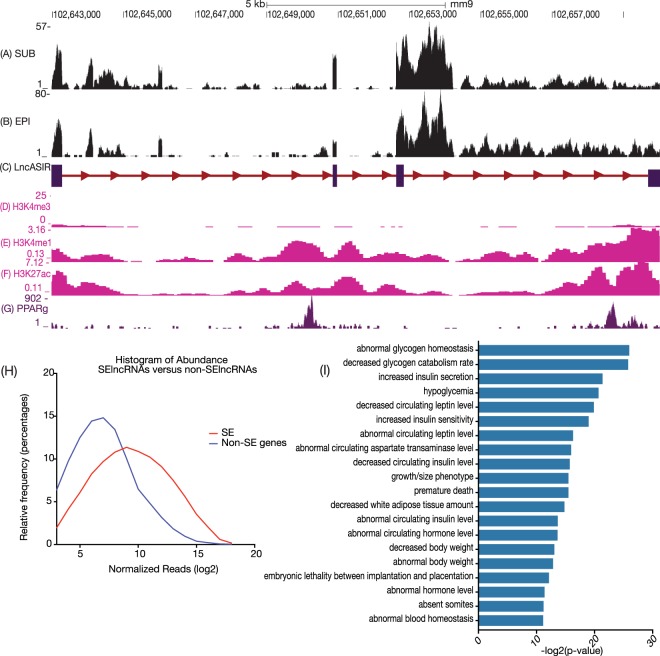
UCSC browser image of LncASIR and super-enhancer epigenetic signature at lncASIR loci. (**A**) RNA-seq data from subcutaneous fat and (**B**) from epididymal fat from adipose tissue lncRNA catalog by Alvarez et al. have been mapped onto UCSC browser. (**C**) Gene structure annotation based on Refseq. (**D**–**F**) ENCODE ChIP-seq signal from H3K4me1, H3K4me3, and H3K27ac marks in brown adipose tissue as density of processed signal enrichment. (**G**) PPARγ binding site from ChIP-seq data. (**H**) Abundance of super enhancer lncRNAs versus non-super enhancer lncRNAs. (**I**) Genomic region enrichment annotation tool result for super enhancer lncRNAs. Graphs have been drawn using Prism 8. While UCSC browser image is generated using UCSC and labels edited using Adobe Illustrator.

The LncASIR was annotated as a 4-exon transcript in the RefSeq database, but the *de novo* assembly from Alvarez *et al*. (Cell Meta, 2015) suggested that LncASIR may have a few different isoforms. Examining the RNA-seq data mapped to the genome in the LncASIR locus, we found multiple clusters of reads across the entire region, supporting the structure annotations from both RefSeq and Alvarez’s study (Fig. 2A–C). Based on the RNA-seq data, quite a few other unannotated transcripts could be generated from this region. Most regions across the LncASIR locus are actively transcribed to generate multiple isoforms.

To understand the complex nature of LncASIR’s structure, we investigated the chromosome architecture in this region. This region bears high-level H3K4me1 and H3K27ac modifications, but low-level H3K4me3 modifications (Fig. 2D–F). Additionally, this region contains multiple PPARγ binding sites (Fig. 2G). Both the chromosome structure and PPARγ binding suggest that LncASIR is transcribed from an enhancer. Reinhardt *et al*. recently reported a list of super-enhancers in adipocytes based on H3K27ac and PPARγ, among which is the LncASIR locus^44^. Thus, LncASIR is a super-enhancer lncRNA.

We further examine whether other super-enhancer lncRNAs are regulated upon insulin treatment. Overlapping lncRNAs with super-enhancers results in the identification of 80 super-enhancer lncRNAs detectable in adipocytes. Super-enhancer lncRNAs tend to be more abundant than lncRNAs derived from non-super-enhancer regions (Fig. 2H) and tend to be located near genes enriched for metabolism pathways (Fig. 2I), supporting an *in cis*-regulatory role of these se-lncRNAs in energy metabolism^45^.

### Silencing LncASIR using dcas9-KRAS and guide RNA in mature adipocyte with insulin treatment

**Figure 3:**
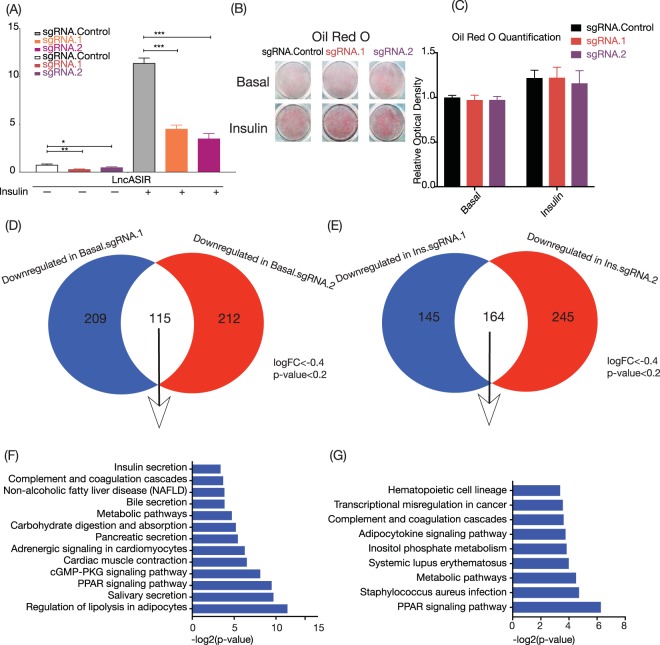
Silencing of LncASIR using dcas9-KRAB lentiviral system in primary white adipocytes. (**A**) Quantitative RT-PCR data for silencing efficiency of LncASIR with and without insulin treatment. (**B**) Oil-Red O staining after knockdown. Representative well have been displayed. (n = 4) (**C**) Semi-quantitative measurement of Oil Red O (n = 4). (**D**) The Venn diagram of the downregulated genes caused by sgRNA.1 and sgRNA.2 in adipocytes at basal level. Threshold for the gene selection: logFC < −0.4 and p < 0.2 (**E**) Same as panel D with insulin treated adipocytes. (**F**,**G**) Pathway analysis for the gene list from panel D and E using DAVID, respectively. Unpaired t-test is used to calculate p-value. *P < 0.05; Error bars are SEM. Graphs have been drawn using Prism 8.

To determine the function of LncASIR in insulin signaling pathway, we conducted a loss-of-function study in cultured primary adipocytes. We failed to achieve satisfactory knockdown of LncASIR with 8 different retroviral shRNA constructs, likely due to LncASIR’s location in the nucleus, so we utilized the dcas9 system to repress the transcription of LncASIR loci. We designed two guide RNAs, expressed them in a lentiviral vector and transduced the lentivirus into pre-adipocytes followed by induction of differentiation for 5 days. Both gRNAs repressed the level of LncASIR by > 60% in cultured adipocytes before and after insulin treatment (Fig. 3A). Impact of silencing on lipid accumulation was measured with or without insulin treatment, cells with insulin treatment showed increased Oil Red O staining while there was no difference between knockdown versus control (Fig. 3B,C).

We performed RNA-seq to examine how silencing LncASIR may affect the insulin-induced gene expression program in adipocytes. Since virus infection may influence cell differentiation efficiency and interfere with many signalling pathways, we first tested whether the infected cells (negative control) are still responsive to insulin treatment. Real-time PCR and RNA-seq analysis indicated a few well established insulin-induced markers including Glut4, Fabp4 and Fasn were still up-regulated upon insulin treatment (Figure S2A,B). The up-regulated pathways are mainly associated with fatty acid metabolism (Figure S2C). Thus, the insulin signalling is mostly intact in our virus-infected cells, which allows us to analyse the effects of LncASIR silence on the insulin-induced program (Figure S3A,B). The influenced genes by two gRNAs significantly overlap (Fig. 3D,E). While genes with an increased expression upon silencing lncASIR resulted in no significant pathway in DAVID (Figure S3C,D), the downregulated genes were associated with metabolic pathways such as PPAR signalling, lipolysis, adipocytokine signalling, inositol phosphate signalling in adipocytes (Fig. 3F,G). Therefore, silencing LncASIR results in a dysregulation of metabolic pathways downstream insulin signalling.

**Figure 4:**
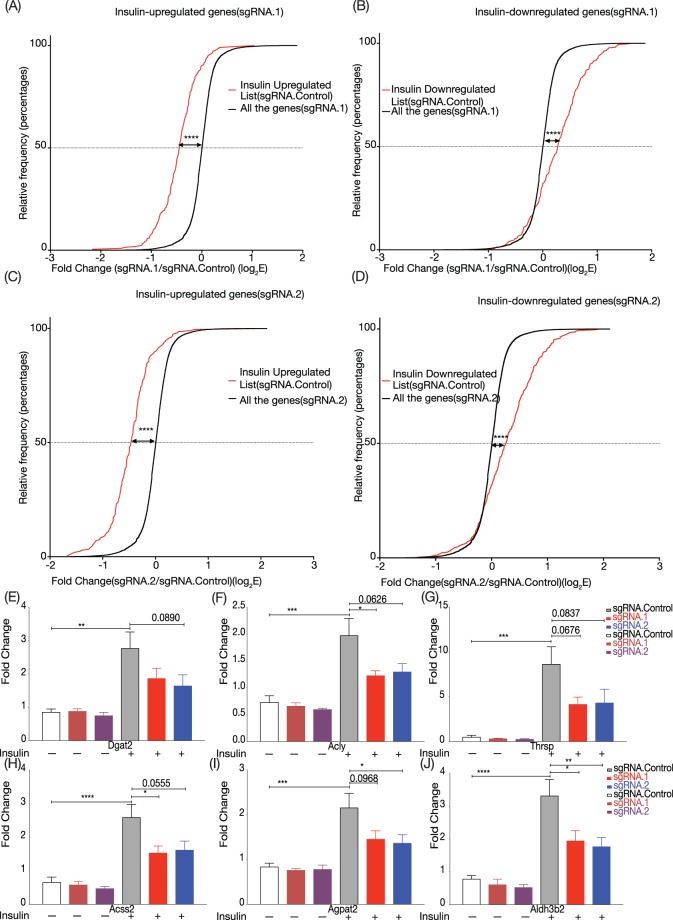
Global shift of insulin responsive genes in adipocytes. (**A**) Cumulative frequency graph of the gene expression fold change caused by sgRNA. for genes that are upregulated in response to insulin. Threshold for gene selection: logFC > 0.67 and p < 0.2. (**B**) Cumulative frequency graph for genes downregulated by sgRNA1. (**C**,**D**) Same as panel A and B for sgRNA.2. Kolmogorov-Smirnov test used to calculate p value between the cumulative curves. (**E–J**) Quantitative RT-PCR validation for metabolism genes whose response to insulin were attenuated by LncASIR repression. Unpaired t-test used to calculate the p values. P < 0.05; *p < 0.005; **p < 0.001; ***. Error bars are SEM. Graphs have been drawn using Prism 8.

While gene ontology analysis was widely used in research, the GO database often lack the cellular context information used for a specific study. To rigorously determine the role of LncASIR in insulin signalling pathway in adipocytes, we compared the genome-wide gene expression in adipocytes before and after insulin treatment to identify up- and down-regulated gene lists. We found that the up- and down-regulated genes were decreased and increased by repression of LncASIR, respectively (Fig. 4A–D). The effects of LncASIR on a few genes including Dgat2, Acly, Thrsp, Acss2, Agpat2 and Aldh3b2 were confirmed by real-time PCR (Fig. 4E–J). Therefore, loss-of- LncASIR results in a significant attenuation of insulin -induced gene program in adipocytes, establishing LncASIR as a new downstream regulator governing transcriptional response to insulin. Similar results were obtained when 30 fold less insulin used (Supplementary Fig. 5A–D). Furthermore, lncASIR was robustly responsive to further dilutions of insulin (Supplementary Fig. 5E).

## Conclusion

LncASIR is a novel factor downstream insulin signalling pathway and controls the adipocytes’ transcriptional response to insulin signalling.

## Discussion

Long noncoding RNAs are new regulators in cellular signal transduction, but their function in insulin signalling pathway was under study in adipose tissue. We have identified an array of lncRNAs that respond to insulin signalling in mature primary adipocytes. One of the long noncoding RNA (LncASIR) is drastically up-regulated in response to insulin. When LncASIR was silenced in insulin-treated adipocytes, the responsive gene program was attenuated, indicating that cells with lower LncASIR do not respond to insulin treatment as well as the control cells. Thus, LncASIR is involved in regulating insulin response in adipocytes.

The mechanism used by LncASIR remains unclear. A recent study from our group showed that lnc-leptin, a lncRNA derived from an enhancer upstream of leptin, could regulate expression of Leptin by governing enhancer-promoter loop formation^37^. Since LncASIR is a super enhancer lncRNA, it could have a function by mediating enhancer-promoter interactions through an *in cis-* or *trans*-chromosomal manner. However, when we cloned and overexpressed this lncRNA in a retroviral vector^35^, we did not detect any significant effect on gene expression (Figure S4). Thus, lncASIR is required for the full insulin-responsive program but not sufficient to induce this program. There are at least two non-mutually exclusive explanations for this phenomenon. Firstly, lncASIR was reported to bind to PRC^46^, it is possible that LncASIR needs to function with its protein partners by forming functional nucleoprotein complexes, but the availability of its protein partners could be limiting. Therefore increasing the expression of lncASIR alone is not sufficient to enhance transcriptional response. Secondly, lncASIR needs to function in its endogenous locus to facilitate enhancer-promoter interactions. However, ectopic over-expression is unlikely to affect the endogenous lncASIR expression, which might explain the lack of functional effects in our overexpression experiment. These hypotheses should be investigated in further studies.

Amount of insulin used to trigger insulin response is higher than the physiological levels as indicated in Ye *et al*.^47^, Tran *et al*.^48^, and Wu *et al*.^49^ while comparable amounts used for measurement of transcriptional response in Grossman *et al*.^50^ and Kang *et al*.^51^. To validate physiological relevance of this experiment, we also performed the same experiment with insulin levels similar to physiological levels and we observed similar changes in insulin downstream genes. Additionally, comparable activation of lncASIR was detected with further dilutions of insulin.

Lack of change in Oil Red O staining and functional adipogenesis could be explained with two underlying reasons. First, knockdown of lncASIR results in an impaired gene response program but did not totally block this program as indicated in Fig. (4E–J). Although the extend of gene expression change by insulin is lower in the knockdown cells, most genes are still regulated, which could be sufficient to support cell differentiation and lipid accumulation. Second, the CRISPRi system significantly reduced the transcription of lncASIR, but there are still 30–40% transcript detectable in the KD cells. The remained fraction of lncASIR could allow adipogenesis and lipid accumulation. This is clearly indicated in Fig. 3A, lncASIR levels in insulin treated knock down is still higher than those in basal control. Thus, presence of lncASIR enough to justify presence of insulin response enough to accumulate comparable amount of lipid droplets in Fig. 3B.

## Methods

### RNA Library Preparation

Total RNA from mature white adipocytes was isolated using a QIAGEN kit. Sequencing libraries were prepared as described^52^ and sequenced on the Illumina HiSeq2000 platform.

### RNA-seq Library Analysis

Raw RNA-seq reads were mapped to mouse genome mm10 with STAR mapping software, and then the raw expression values were identified using FeatureCounts based on the GENCODE annotation. Afterwards, normalized and differentially expressed genes analysis were performed by DESeq2. By using the gene type annotation from GENCODE, all expressed genes were further separated to lncRNAs and mRNAs.

### Knockdown Using Dcas9-KRAB Silencing

Pre-adipocytes at ~80% confluence were infected with lentivirus expressing sgRNAs and cas9-KRAB construct. ~16 hr later, cells were recovered in full culture medium, induced to differentiate 48 hr post-infection. Dcas9-KRAB-sgRNA knockdown in preadipocytes was performed as described^53^. Oligoes used in this study are listed in Table S1.

### Primary Adipocyte Isolation and Adipocyte Differentiation

Stromal vascular cells isolation and adipocyte differentiation are performed as described^52^. Briefly, harvested inguinal adipose tissues from 3- to 4-week-old B/C mice were washed, minced, and then digested for 25 min at 37 °C, with brief vortex every 5 minutes in collagenase(0.2%). Digestion was stopped with DMEM and cells were filtered through 100 μm strainer. Cells were collected by centrifuge (1000 rpm for 5 minutes). The supernatant was removed. Isolated SVF cells were then plated and cultured in 10% CO_2_ at 37 °C in DMEM with 10% fetal bovine serum(FBS, Invitrogen), 50 units/mL penicillin, 50 mg/mL streptomycin, and 10 mg/mL gentamicin (Invitrogen). Cells were grown till confluency in DMEM with 10%FBS. For adipocyte differentiation, confluent cultures were exposed to the adipogenic cocktail supplemented with dexamethasone (0.5 μM, Sigma), insulin (0.85 μM, Sigma), isobutylmethylxanthine (250 μM, Sigma), Rosiglitazone (1 μM, Sigma) in DMEM with 10% FBS for 48 h. After 48 h, cells were maintained in DMEM with 10% FBS containing insulin (0.17 μM) for 48 h. Cells were maintained in DMEM with 10% FBS for 48 hours before treatment. Post differentiation, cells were fasted for 6 hours in FBS free DMEM and then treated with insulin (1.72 μM) for 8 hours to induce transcriptional change. Insulin concentration for the treatment chosen since it is similar to previous transcriptional analysis assays amount from the literature.

### Plasmid and Retroviral Transduction

sgRNA viral plasmids and dcas9-KRAB plasmid were co-transfected with lentiviral packaging vectors pMD2.g(Addgene #12259) and pSPAX2(Addgene #12260) and into 293 T cells using FuGENE6 (Promega). After the overnight, media was replaced with fresh media. Viruses were collected 48 hr post-transfection. Pre-adipocytes were infected by fresh virus in the presence of polybrene (8 ng/ml final concentration) for overnight and replenished with fresh medium. Cells were induced to differentiate 48 hr post-infection and collected for downstream analysis at the indicated times.

### Oil Red O Staining and Quantifications

Media was aspirated from the cells, and cells were washed with PBS. 10% formalin was added to the cells 1 hour at room temperature. Formalin was removed, and cells were rinsed with 60% isopropanol. After aspirating isopropanol, working Oil Red O solution was added on top of the cells for one hour. Oil Red O was removed, and cells were washed with water. Pour off distilled water and allow dishes to air dry. Cell culture plate was scanned to take image. To quantify the lipid abundance, 200ul of isopropanol was added to wash the stain in each well of a 24-well plate. The lipid contents in each well were reflected by measuring the absorbance at OD_500_.

### Genomic Region Enrichment Annotation Tool (GREAT)

GREAT^45^ is an available online tool that can provide insights about lncRNA function *in cis* by measuring the enrichment of genes within 500 kb of a provided region. In our analysis, we provided the transcription start and end sites of lncRNAs. Further details are available on http://great.stanford.edu/public/html/index.php.

### Animal Work Ethical Approval

Animal work has been approved under IACUC-1179 in DUKE-NUS Graduate Medical School to Dr. Sun Lei Lab. All experiments were performed in accordance with the relevant guidelines and regulations.

### Data Accession and Supplementary Information

ChIP-seq data for histone markers are available on ENCODE website. PPARγ ChIP-seq is available through previously a published article by Rajakumari *et al*.^54^. Supplementary files contain insulin responsive gene list, insulin responsive lncRNAs, insulin responsive super enhancer lncRNAs and oligoes/primers used in this project.

## Supplementary information

Supplementary Figures

Insulin Response Gene List

Table 1 (Primers and oligoes)

Insulin Responsive lncRNA list

Insulin Responsive Super Enhancer lncRNAs in Adipose Tissue

## Data Availability

Key gene lists has been provided as supplementary excel files. Raw data and remaining datasets generated during and/or analysed during the current study are available from the corresponding author on reasonable request.
