# Supplementary material for: Silencing an insulin-induced lncRNA, LncASIR, impairs the transcriptional response to insulin signalling in adipocytes
Source: Sci Rep. 2019 Apr 4;9:5608. doi: 10.1038/s41598-019-42162-5 (PMC6449399; doi:10.1038/s41598-019-42162-5)
Supplement: Supplementary file 1 — Supplementary Figures [file 41598_2019_42162_MOESM1_ESM.pdf]

Silencing an insulin-induced lncRNA, LncASIR, impairs the transcriptional response to insulin signalling in adipocytes

Ufuk Degirmenci[2,3], Jia Li[1], Yen Ching Lim[1], Diana Teh Chee Siang[1], Shibo Lin[4], Hui Liang[4,*], Lei Sun[1,2,*]

Affiliations;

[1]Cardiovascular and Metabolic Disorders Program, Duke-NUS Graduate Medical School, 8 College Road, Singapore 169857, Singapore

[2]Institute of Molecular and Cell Biology, Agency for Science, Technology and Research, 61 Biopolis Drive, Proteos, Singapore 138673, Singapore

[3]Department of Biological Sciences, National University of Singapore, Singapore 117558, Singapore

[4]Department of General Surgery, the First Affiliated Hospital of Nanjing Medical University, Nanjing, 210029, China

* Corresponding Authors;

Email; Hui Liang: drhuiliang@126.com; Sun Lei: sun.lei@duke-nus.edu.sg

Supplementary Figure 1

(A)

	Reads
Basal.1	79113134
Basal.2	58062089
Insulin.1	78038179
Insulin.2	76325521

(B)

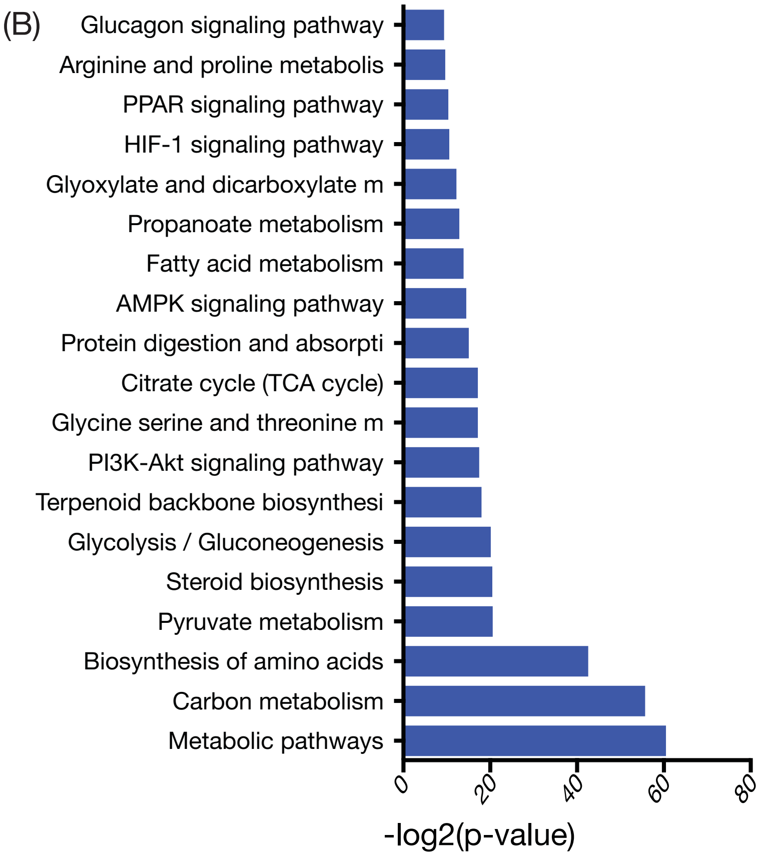

Supplementary 1: (A) Number of reads from RNA-seq for Figure 1A and Figure 1B. (B) KEGG pathway analysis for the differentially expressed genes upon insulin treatment.

Supplementary Figure 2

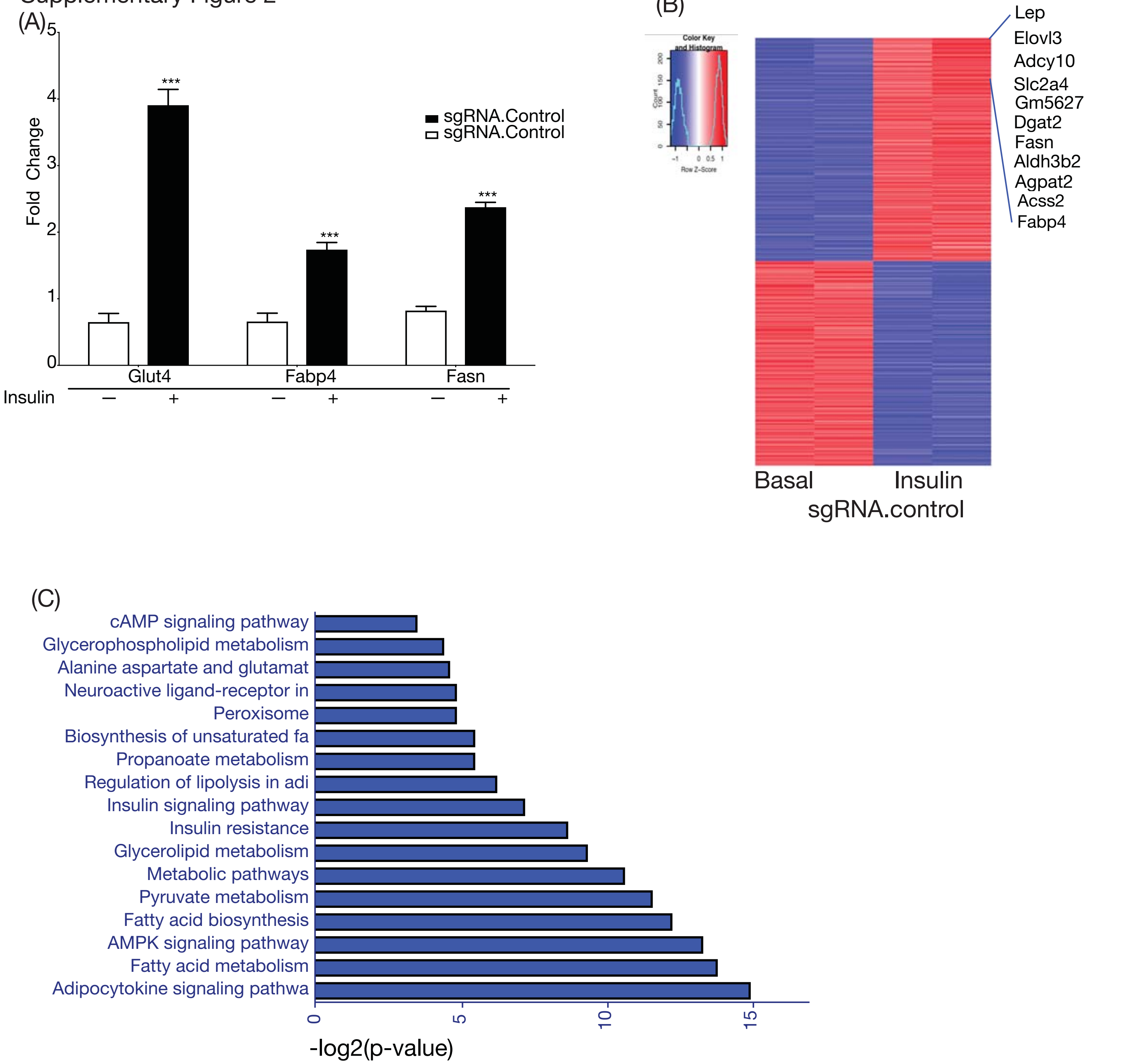

Supplementary 2: (A) Quantitative RT-PCR for major insulin downstream genes from sgRNA.control t-test used for p values. (B) Heatmap from sgRNA.control for differentially expressed mRNAs. Top mRNAs are shown on the side as reference. (C) KEGG pathway analysis for differentially expressed genes from sgRNA.control. Heatmaps were drawn using R. Bar graphs were drawn using Prism 8. KEGG analysis have been performed using DAVID.

Supplementary Figure 3

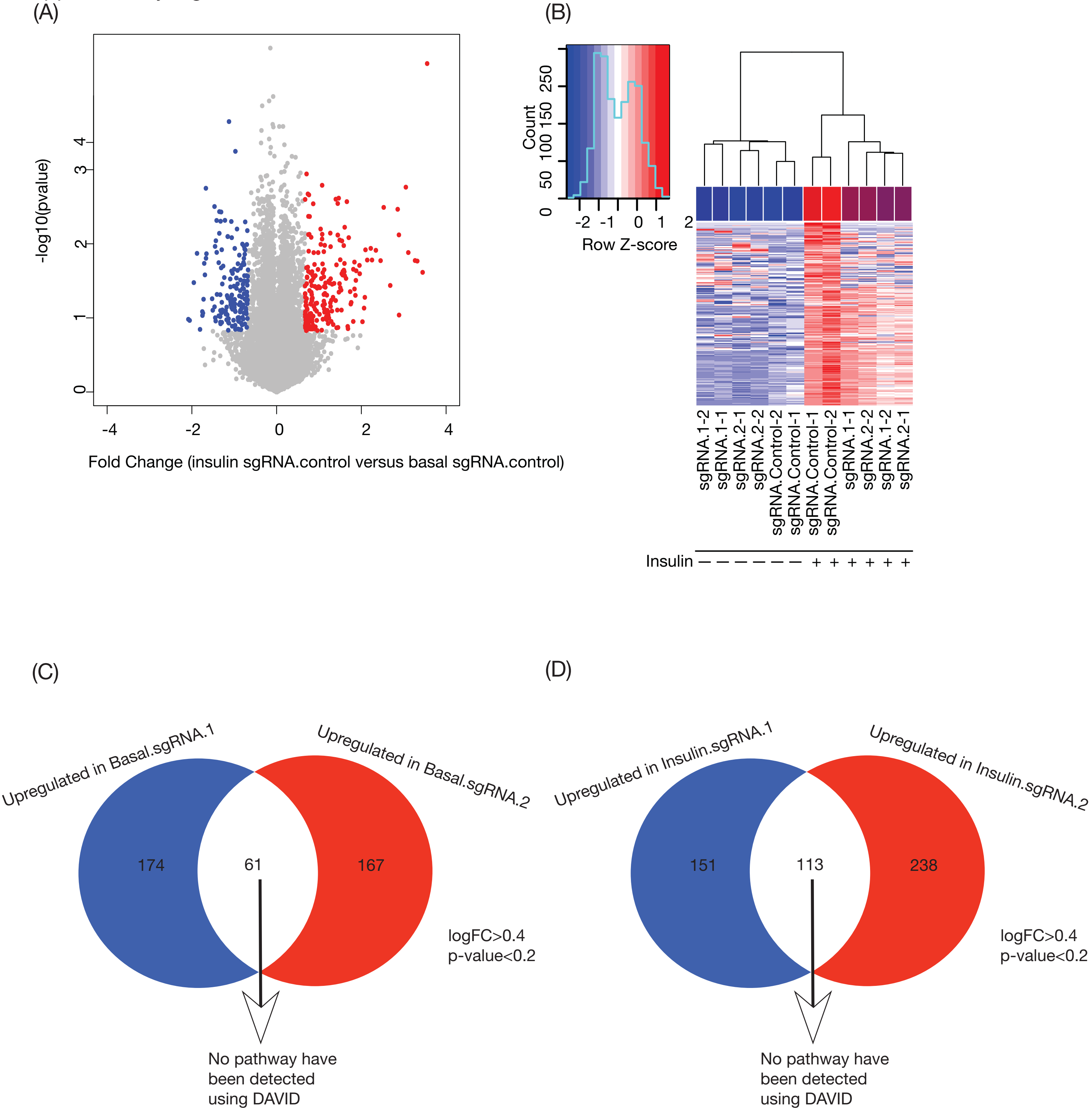

Supplementary 3: (A) Volcano graph of fold change versus p value from insulin treated control versus basal control. (B)Heatmap for insulin upregulated genes with all RNA-seq samples. (C) Overlapping upregulated genes for basal level from sgRNA.1 and sgRNA.2 (D) Overlapping upregulated genes for insulin treated levels from sgRNA.1 and sgRNA.2. No KEGG pathway was detected using DAVID.

Supplementary Figure 4

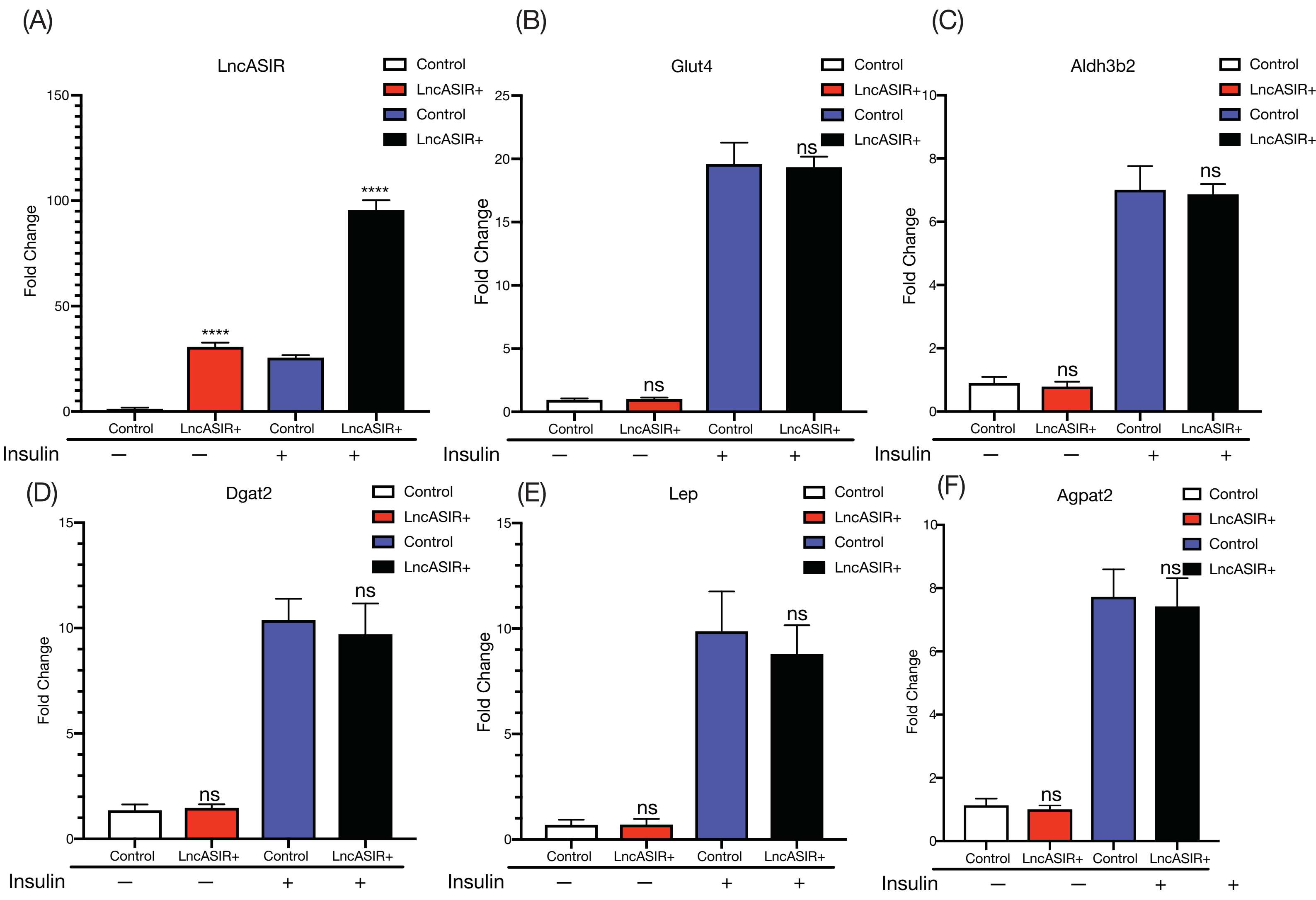

Supplementary 4: Overexpression of LncASIR in mature adipocytes. (A) LncASIR was overexpressed in primary adipocytes using retroviral delivery at preadipocytes and cells were differentiated. At day 5, RNA was isolated and Quantitative RT-PCR has been performed. Insulin treatment was performed similar to figure 1. Without insulin, there was ~40 fold increase of LncASIR. Similar increase was observed within insulin treat group as well. (B-C-D-E-F) Expression of insulin downstream genes upon overexpression from Quantitative RT-PCR. Unpaired t-test is used to calculate p-value. Ns: non-significant. RPL23 was used to calculate CT. Graphs were drawn using Prism 8.

Supplementary Figure 5

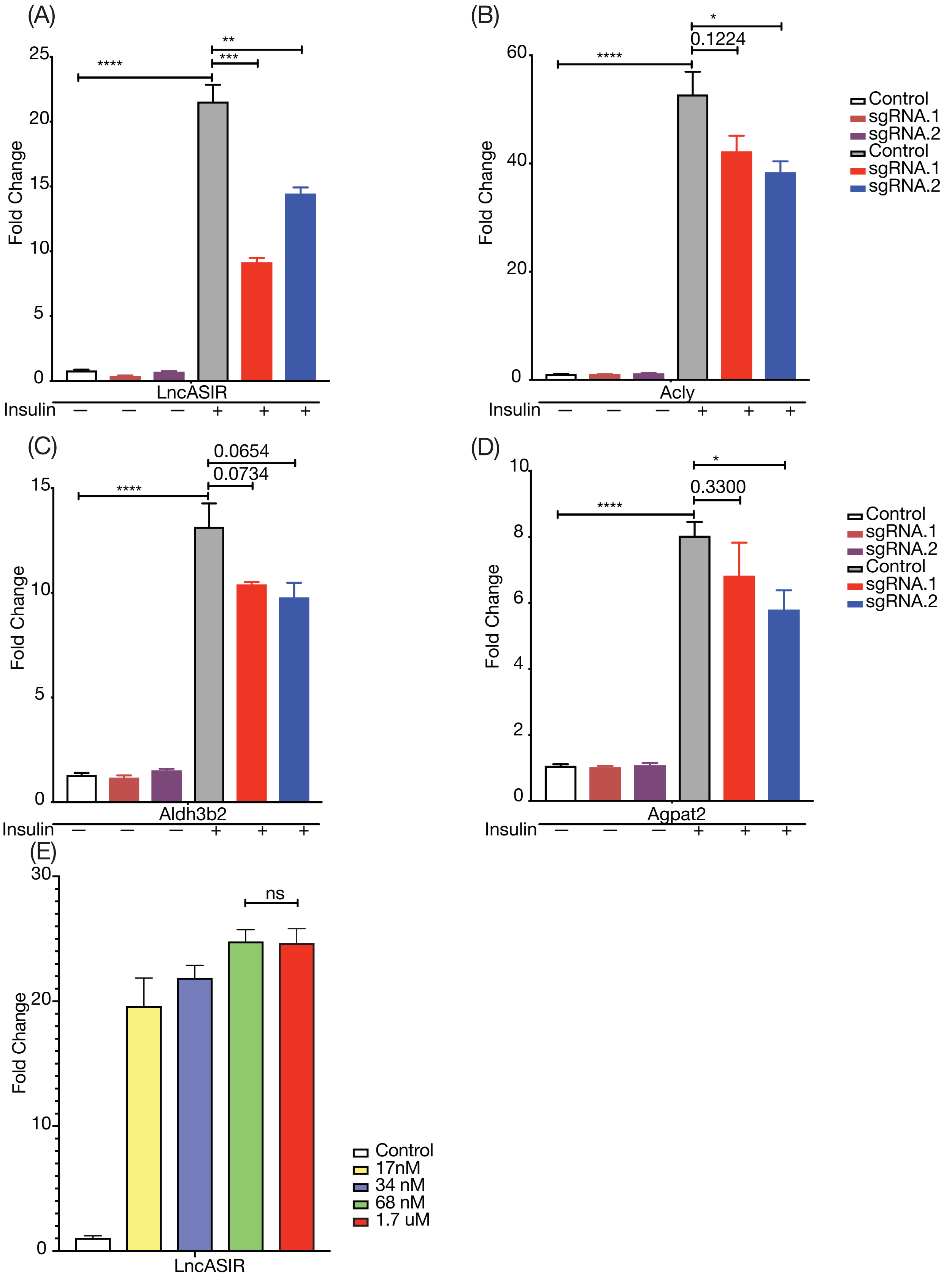

Supplementary 5: Silencing of LncASIR with 30-fold less insulin treatment (56 nM). (A) Knockdown of LncASIR with or without insulin. Experiment from figure 1 was repeated and Quantitative RT-PCR has been performed. (B-C-D) Subset of genes from figure 4 used to check the downstream impact. Unpaired t-test is used to calculate p-value. P<0.05; *. RPL23 was used to calculate CT. Graphs were drawn using Prism 8. (E) Varied concentrations (1.7 M, 68 nM, 34nM and 17 nM) of insulin showed comparable amount of transcriptional activation of LncASIR with no significant difference between 1.7 M and 68 nM.
